# Image quality evaluation for a clinical organ-targeted PET camera

**DOI:** 10.3389/fonc.2024.1268991

**Published:** 2024-03-25

**Authors:** Brandon Baldassi, Harutyun Poladyan, Anirudh Shahi, Henry Maa-Hacquoil, Madeline Rapley, Borys Komarov, Justin Stiles, Vivianne Freitas, Michael Waterston, Olexiy Aseyev, Alla Reznik, Oleksandr Bubon

**Affiliations:** ^1^ Department of Physics, Lakehead University, Thunder Bay, ON, Canada; ^2^ Radialis Inc., Thunder Bay, ON, Canada; ^3^ Department of Medical Imaging, University Health Network, Sinai Health System, Women’s College Hospital, Toronto, ON, Canada; ^4^ Department of Medical Imaging, University of Toronto, Toronto, ON, Canada; ^5^ Department of Medical Oncology, Thunder Bay Regional Health Sciences Center, Thunder Bay, ON, Canada; ^6^ Thunder Bay Regional Health Research Institute, Thunder Bay, ON, Canada

**Keywords:** organ-targeted PET, PET, PEM, NST, recovery coefficient

## Abstract

**Introduction:**

A newly developed clinical organ-targeted Positron Emission Tomography (PET) system (also known as Radialis PET) is tested with a set of standardized and custom tests previously used to evaluate the performance of Positron Emission Mammography (PEM) systems.

**Methods:**

Imaging characteristics impacting standardized uptake value (SUV) and detectability of small lesions, namely spatial resolution, linearity, uniformity, and recovery coefficients, are evaluated.

**Results:**

In-plane spatial resolution was measured as 2.3 mm ± 0.1 mm, spatial accuracy was 0.1 mm, and uniformity measured with flood field and NEMA NU-4 phantom was 11.7% and 8.3% respectively. Selected clinical images are provided as reference to the imaging capabilities under different clinical conditions such as reduced activity of 2-[fluorine-18]-fluoro-2-deoxy-D-glucose (^18^F-FDG) and time-delayed acquisitions. SUV measurements were performed for selected clinical acquisitions to demonstrate a capability for quantitative image assessment of different types of cancer including for invasive lobular carcinoma with comparatively low metabolic activity. Quantitative imaging performance assessment with phantoms demonstrates improved contrast recovery and spill-over ratio for this PET technology when compared to other commercial organ-dedicated PET systems with similar spatial resolution. Recovery coefficients were measured to be 0.21 for the 1 mm hot rod and up to 0.89 for the 5 mm hot rod of NEMA NU-4 Image Quality phantom.

**Discussion:**

Demonstrated ability to accurately reconstruct activity in tumors as small as 5 mm suggests that the Radialis PET technology may be well suited for emerging clinical applications such as image guided assessment of response to neoadjuvant systemic treatment (NST) in lesions smaller than 2 cm. Also, our results suggest that, while spatial resolution greatly influences the partial volume effect which degrades contrast recovery, optimized count rate performance and image reconstruction workflow may improve recovery coefficients for systems with comparable spatial resolution. We emphasize that recovery coefficient should be considered as a primary performance metric when a PET system is used for accurate lesion size or radiotracer uptake assessments.

## 1 Introduction

The diagnostic capabilities of organ-targeted Positron Emission Tomography (PET) systems depend on the ability to reconstruct the true radiotracer activity within a lesion and on the conspicuity of small lesions at different injected activities. The former is of particular importance in evaluating response to neoadjuvant systemic treatment (NST) in breast cancer patients - chemotherapy or hormonal therapy administered prior to surgical treatments (1). Neoadjuvant treatment is increasingly being used to downstage and downsize tumors before resection surgery and thus to facilitate breast conservation. Early and accurate assessment of the tumor’s response to NST (i.e., the metabolic decline and the reduction in size) can help to determine a personalized treatment regimen to achieve optimal response prior to the surgery and to avoid the toxicity associated with ineffective treatments. A decline in tumor metabolism in response to NST can occur earlier than apparent changes in tumor size and anatomy [or may not correlate with anatomical changes at all (2)], thus making anatomical imaging modalities not well suited for the purpose of evaluating early treatment response. In contrast, PET molecular imaging with ^18^F-fluoro-2-deoxyglucose (^18^F-FDG) may better reflect early treatment response by its ability to detect a decrease in tumor glucose metabolism preceding a decrease in its anatomical size (3, 4).

Recent trends to use NST for tumors smaller than 2 cm in size (5) have put a stringent requirements on PET/CT (Computed Tomography) performance for quantitative assessment of the metabolic changes in tumors through measurements of standardized uptake value (SUV). In fact, when ^18^F-FDG uptake in small tumors is measured, the partial-volume effect (PVE), a consequence of finite spatial resolution, can lead to underestimation of activity concentrations in reconstructed PET images due to spill-over of counts between different regions within the image (6). The PVE becomes significant for an imaging system when the dimensions of a tumor are less than two to three times the full width half maximum (FWHM) point spread function (PSF) of the system (7), as this can strongly influence the determined size and uptake of the lesion. Therefore, with 6 mm spatial resolution of most modern PET/CT scanners, PVE can affect SUV measurements and activity reconstruction in shrinking tumors that were around 2 cm prior to treatment. This may produce inaccuracies in assessing response to neoadjuvant treatment: a shrinking tumor will look larger but less aggressive than it really is due to signal spill-over from lesion-to-background and degradation of recovery coefficient. Alternatively, if NST results in partially necrotic centers within tumors, signal spill-in will falsely indicate a greater extent of viable tissue within the inactive parts of the tumor than in reality. The PVE is quantitatively assessed by the ratio between image-derived and true activity measurements, commonly termed the recovery coefficient (RC), and depends on several factors which include the spatial resolution, the count rate efficiency, and the reconstruction algorithm and parameters (8).

The development of a high-sensitivity organ-targeted Positron Emission Tomography (PET) system – the “Radialis PET camera” – has developed from the clinical need to reduce the radiation dose associated with functional (molecular) imaging while preserving the small lesion detection capability inherent to organ-targeted PET (9–12). We have recently demonstrated that the Radialis PET camera has improved sensitivity, capable of significant dose reduction (factor of 10) in comparison to commercial whole-body (WB) PET scanners (12). Standardized measurements were performed with NEMA NU-4 procedures adapted for the planar PET detector geometry, including spatial resolution, sensitivity, and system count rates. Selected clinical breast cancer images shown below illustrate the system performance within a range of conditions including varied radiation doses (37-370 MBq), the presence of chest wall lesions, and lesion detectability in comparison to WB-PET, full field digital mammography (FFDM), and breast MRI. The increased sensitivity shown by the NEMA NU-4 tests and the high-efficiency radiotracer detection demonstrated with the clinical images were achieved by the development of a new type of modular detector architecture with four-side tileable sensor modules based on high-gain Silicon Photomultiplier (SiPMs) photosensors (13).

Standardized measurements within NEMA NU-4 are important to compare the Radialis PET camera to similar devices, however these standards were developed without consideration of the latest hardware and software developments and therefore have faced recent criticism (14). Indeed, the NEMA NU-4 requirement of back-projection image reconstruction does not represent the methods used in current real-world, clinical applications. Therefore, the described tests have potential flaws in accurately representing the system performance metrics during typical use (12, 14). In addition, since NEMA NU-4 standard tests were developed for preclinical imaging, they do not account for unique aspects of clinical organ-targeted PET [e.g., relatively large field-of-view (FOV)] and detector architectures, including planar detectors and modular, adjustable gantry. Finally, the NEMA NU-4 phantom imaging conditions distinctly differ from clinical use and do not provide needed insights into true clinical capabilities and limitations.

A comprehensive assessment of imaging performance in organ-targeted PET requires additional tests that characterize imaging parameters not covered by NEMA NU-4 standard and which are more suitable for the intermediate FOV and modern iterative image reconstruction methods. Therefore, we follow the methodology developed and reported by others (11, 15, 16) to perform characterization of spatial resolution and linearity, flood field uniformity, and RC, with evaluation of NEMA NU-4 image quality phantom also included. The ability to recover the activity of small structures in the presence of background radioactivity is assessed using micro-spheres of different sizes in a hot background which mimic lesions in the body. The tests of RC, flood field uniformity, contrast to noise ratio (CNR) and the Rose Criterion (17) are of importance for assessing the ability of the system to apply SUV analysis to lesions of different size and for assessing uptake of a radiopharmaceutical.

Additionally, the modular design of our system may allow variability in the electronic function between different modules. This variability may cause spatial distortions along the FOV, as well as non-uniformity between different modules. Thus, experiments with line sources (rather than point sources used in NEMA NU-4) and large-area flood phantoms can serve to better identify any discrepancies in spatial resolution, signal to noise ratio, and uniformity within the entire image space.

Finally, we present selected clinical images with quantification of SUV and 3-D visualization of abnormal metabolic tissue. The measurements reported here provide a performance assessment of the Radialis PET camera, highlighting its capabilities for quantitative PET imaging.

For a variety of emerging clinical applications, the assessment of the size and activity uptake in a lesion is not less important than detection of the lesion itself. *While spatial resolution is one of the main specifications that is used to characterize PET system performance, high spatial resolution is a required but not a sufficient criterion for accurate contrast recovery.* We emphasize that systems with the same or comparable spatial resolution may report different recovery coefficients and different spill-over ratios. Here, we provide PET system performance metrics measured with standardized NEMA protocols, as well as adapted tests used by others (11, 15, 16) and discuss the differences in system performances with special emphasize on recovery coefficient in small lesions.

## 2 Materials and methods

**Figure 1:**
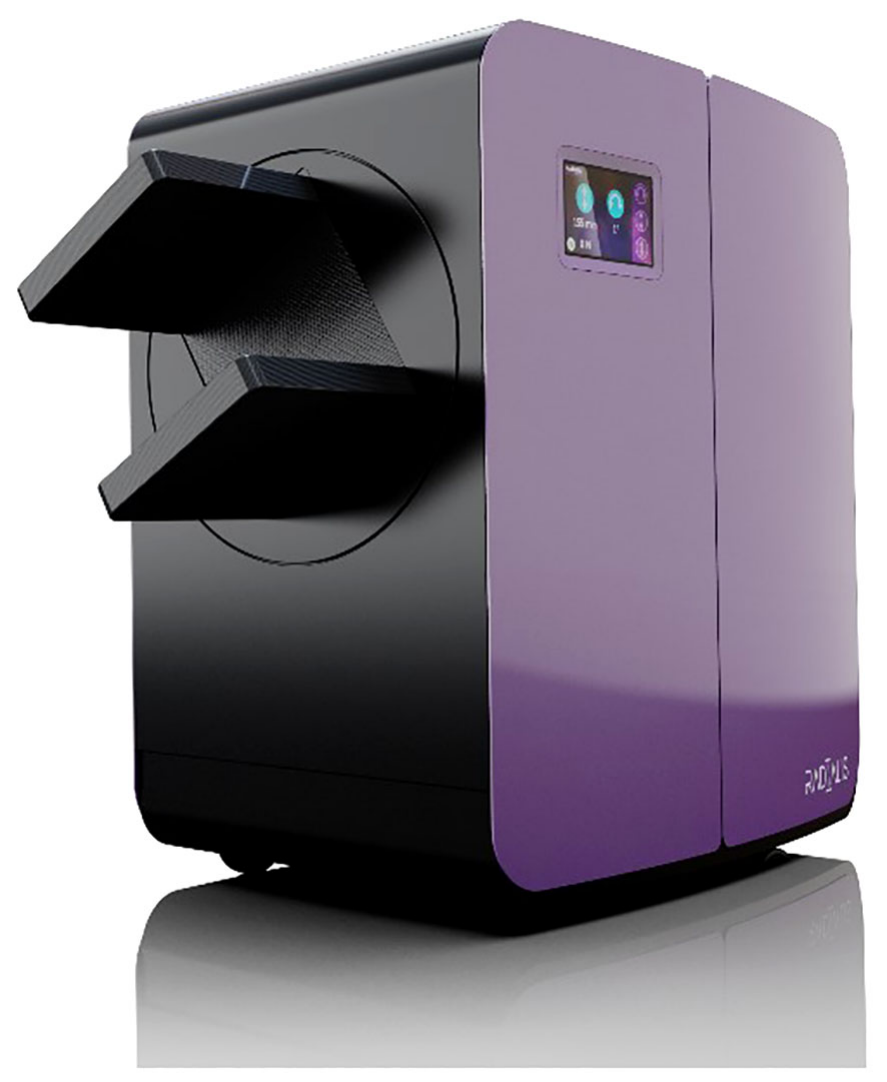
Configuration of the organ-targeted PET Camera with two planar detector heads for positioning on either side of an organ.

**Figure 2:**
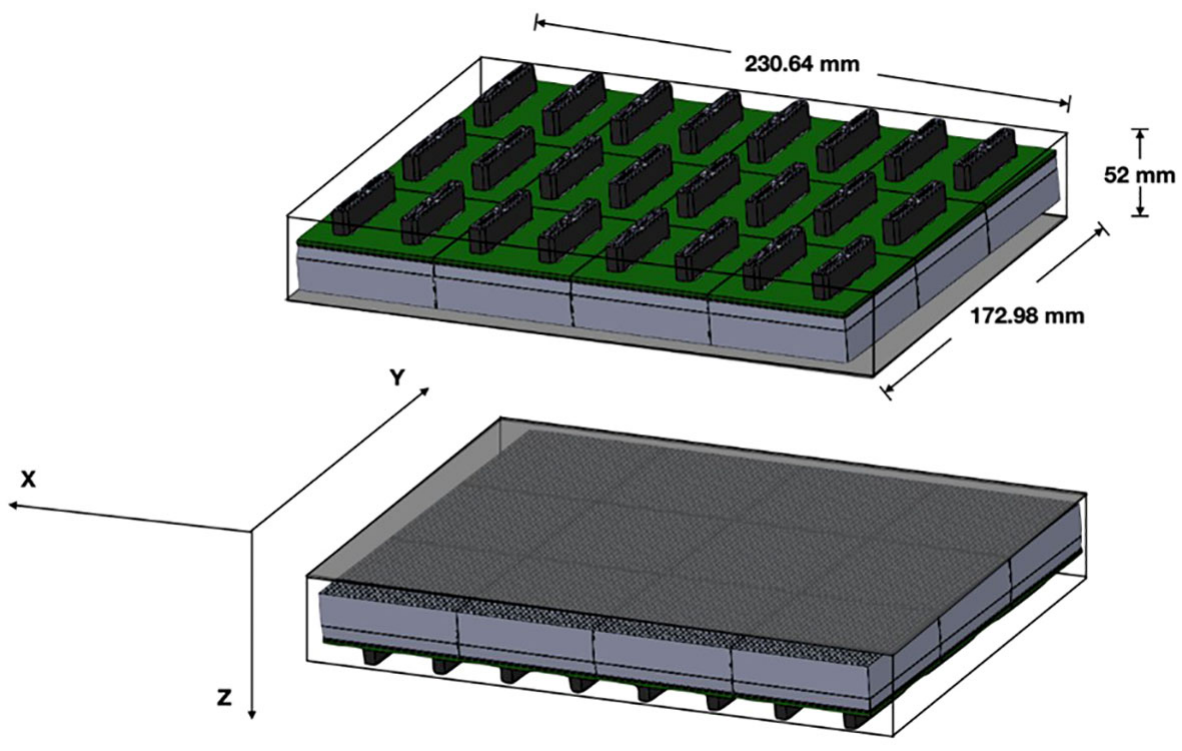
Detector schematic showing the overall size of the detector heads with 3x4 array of sensor modules per detector head and the axis convention for measurements.

The organ-targeted PET camera described herein utilizes an adjustable and versatile planar detector configuration for imaging different organs including the breast, prostate, and heart. The device employs two planar detector heads mounted on a movable gantry (**Figure 1**). Each detector head contains 12 four-side tileable (mosaic) sensor modules that are arranged against each other in a 3x4 array (12) to assemble a seamless, uniform sensing area measuring ~230 mm × ~173 mm (**Figure 2**). The detector heads are enclosed in a thin housing which permits the active imaging area to be just 4 mm from the housing edge. The adjustable gantry permits positioning of the detectors proximal to the organ of interest. Detailed information on the Radialis PET technology can be found in reference (12).

The timing and energy windows for image acquisitions are set at 4 ns and 350-700 keV respectively. Detector separation was configured according to the clinical case or phantom dimensions and ranged between 60 - 135 mm. An iterative maximum likelihood expectation maximization (MLEM) algorithm with 15 iterations is used for image reconstruction. The number of iterations was selected to optimize visualization of small objects while maintaining acceptable flood field uniformity. Default clinical reconstruction parameters are applied to all images unless otherwise specified, including median root prior (MRP) filter (18), attenuation and scatter correction, and solid angle allowance filter (12). Reconstructed images are saved in DICOM format with 24 axial image slices in the XY plane. The image matrix is defined by a pixel size of 0.4 mm × 0.4 mm. The voxel dimension is determined by the detector separation divided into 24 equal components and may vary among acquisitions.

### 2.1 Spatial resolution & linearity

**Figure 3:**
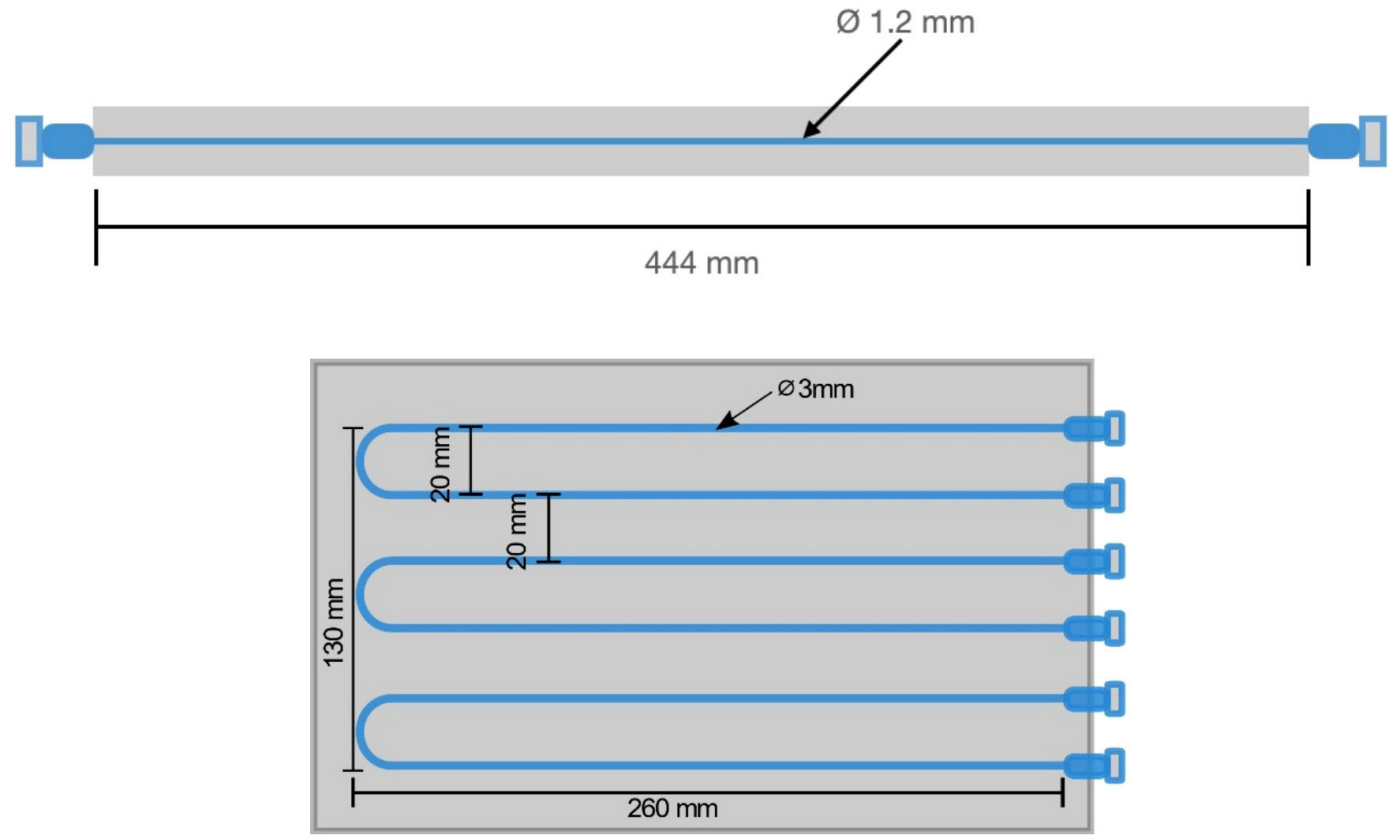
Schematic diagram of the fillable capillary phantom (top) and spatial linearity phantom (bottom) with markings for the line source separation and phantom dimensions.

The spatial resolution measurement was previously conducted using a Na-22 point source in accordance with pre-clinical NEMA standards (12). Here, the spatial resolution is assessed following whole-body PET (WB PET)standards by analyzing the line-spread function (LSF) of a line source of radioactivity in **Figure 3** (19–21). LSF offers a more comprehensive characterization of system performance as it accounts for the entire spatial response profile. This approach provides a more accurate representation of spatial resolution, particularly for organ-targeted PET systems with comparatively large fields of view (FOV).

A capillary tube, with a length of 44.4 cm and an inner diameter of 1.2 mm (which is approximately half of the anticipated spatial resolution), was filled with an ^18^F-FDG solution. The line source was positioned halfway between the detectors axially and centrally in the y-axis, such that the source extends along the entire length of the x-axis FOV. Detected coincidence events are collected until at least 1 million events are recorded. The reconstructed image is analyzed by taking the LSF orthogonal to the line source axis. A gaussian fit is applied and the full width at half maximum (FWHM) for the LSF defines the spatial resolution quoted here (22). LSF’s were taken in 10 positions across the line source spanning the complete FOV. The average value of the FWHM was reported as the spatial resolution for the in-plane and cross-plane FOVs.

Spatial linearity was measured with a linearity phantom shown in **Figure 3**. Six capillary tubes were filled with ^18^F-FDG solution and arranged in a plastic jig to ensure parallel positioning with a center-to-center distance of 20 mm.

Image acquisition for the linearity phantom was performed in two different positions:

1. In the central XY plane (z = 0 mm), with capillaries parallel to y-axis.
2. In the central XY plane (z = 0 mm), with capillaries parallel to x-axis.

Measurements of spatial linearity are derived from pixel values perpendicular to the length of the capillary tubes. The peak pixel value location was determined for each parallel capillary and the separation between each peak was plotted. Variation in reconstructed peak position from known spacing is reported. The spatial accuracy of the reconstructed source is quantified as the difference in average reconstructed position from expected position. The acquisitions were performed at a detector separation of 100 mm with the phantom centered between detectors.

### 2.2 Flood field uniformity

Measurement of flood field uniformity was performed using a flat phantom which is dimensionally greater than the FOV in order to assess imaging effects at the edge of the FOV (23, 24). The phantom was filled with 100 µCi ^18^F-FDG activity and was positioned parallel to and equidistant from each detector. Image acquisition of at least 5 million coincidence events was performed with a detector separation of 80 mm. The image of the flood phantom was reconstructed with the default clinical parameters, using images from the first iteration and fifteenth iteration for analysis. A central ROI of 150 mm × 100 mm was chosen within which the statistical measurements were performed.

Measurements are reported for the mean, maximum, and minimum pixel value, and percentage standard deviation (%STD) as a measurement of noise. These values were calculated based on the methods described for determining the uniformity of the NEMA NU-4 small animal phantom (25) and are further explained in section (D) below. The uniformity analysis was performed and used for per pixel efficiency corrections.

### 2.3 Recovery coefficients

**Figure 4:**
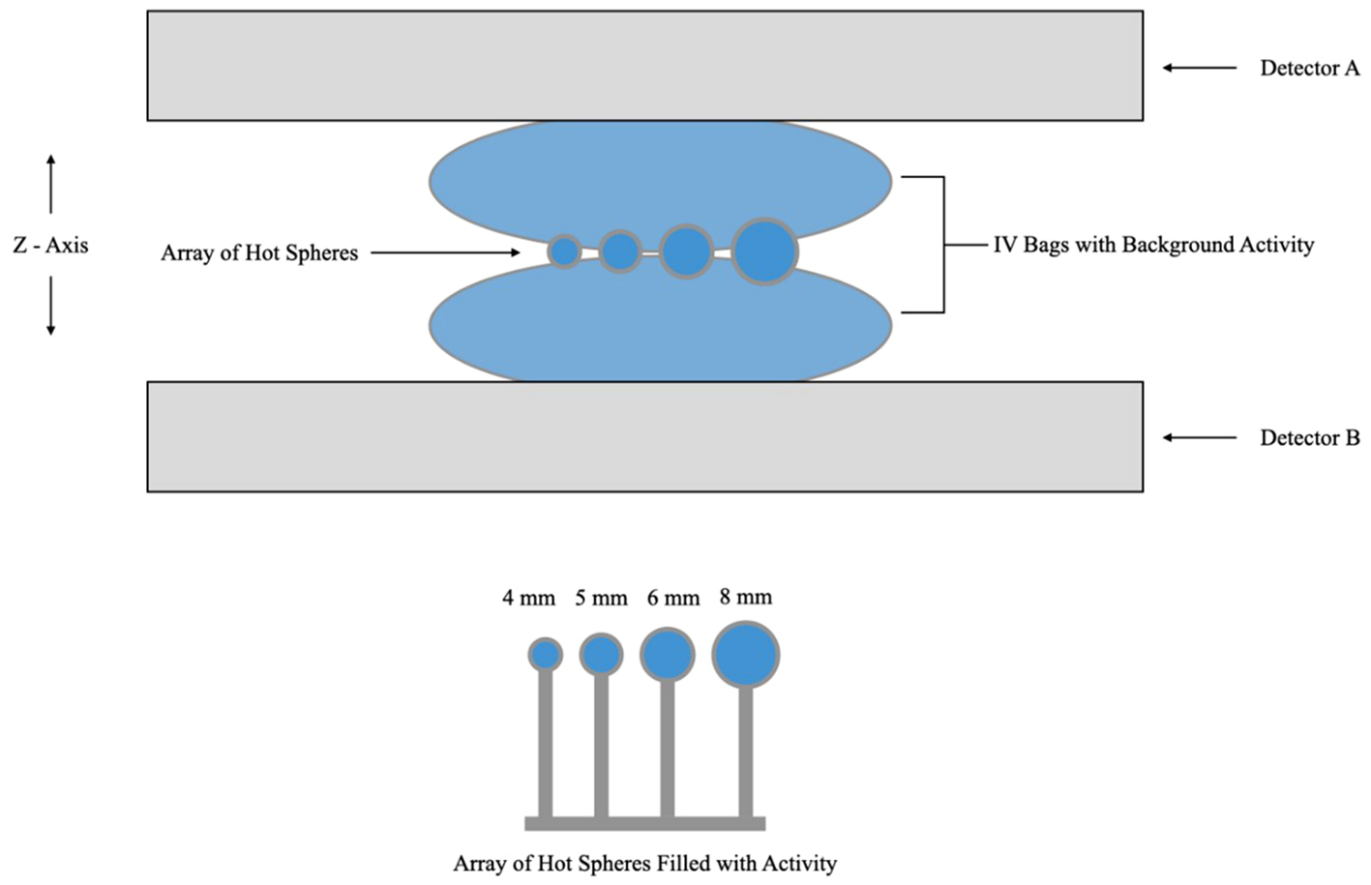
Schematic diagram of the acquisition layout for the recovery coefficient experiment showing hot spheres of radioactivity positioned between two IV bags for background activity and immobilized between detector heads. Note that the schematic is not to scale.

We compared RC with 4:1 and 10:1 lesion to background activity concentrations for, Radialis PET, PEM Flex Solo II (11) and MAMMI PET (16) commercial organ-dedicated PET scanners. Measurements were performed using micro-spheres of radioactivity placed between two 500 mL IV bags filled with background activity. The acquisition layout is presented in **Figure 4**. The spheres, with inner diameters of 4, 5, 6, and 8 mm, were each filled with the same activity concentration of ^18^F-FDG. The IV bags were also filled with ^18^F-FDG activity. Activity concentration of the background was 5 kBq/mL and 0.379 kBq/mL, with sphere activity concentrations of 20 kBq/mL and 3.79 kBq/mL, respectively. Image reconstruction was performed with the default clinical parameters. Detector separation was set to 90 mm to provide slight compression to the IV bags and to mimic clinical imaging conditions, where radioactive tissue is in contact with the detector surface. It should be noted that measurements for the PEM Flex Solo II scanner were performed with a similar experimental configuration as in **Figure 4**, with hot spheres of radioactivity sized from 8 mm to 30 mm between background activity at a 4:1 ratio with background activity of 5kBq/mL (11). Measurements for the MAMMI PET were performed using a cylindrical phantom with hot cylinders in uniform background activity at a 10:1 ratio with background activity concentration of 6 kBq/mL and hot cylinders filled with 58 kBq/mL activity concentration (16).

RCs for each micro-sphere were defined as relative and absolute measures. Relative RCs (Equations 1 and 2) give the ratio between measured pixel values for hot-spheres and background IV bag regions, while absolute RCs (Equation 3) relate the measured activity concentration values to the true activity concentrations measured by a dose calibrator. Maximum image intensity values were measured within a circular region of interest (ROI) around each sphere and the mean image intensity values are calculated within a circular ROI proportional to the sphere diameter and in the uniform part of the IV bag for background. These values were recorded for each sphere in the image and plots were created for the recovery coefficients as a function of sphere diameter and activity concentration.


(1) $$relative\;RC_{mean}=\frac{mean\;\left(hot\;sphere\;ROI\right)}{mean\;\left(background\right)}$$

(2) $$relative\;RC_{max}=\frac{maximum\;\left(hot\;sphere\;ROI\right)}{mean\;\left(background\right)}$$

(3) $$absolute\;RC_{\max}\;=\;\frac{maximum\;(activity\;concentration\;ROI)}{true\;(activity\;concentration\;ROI)}$$

The percent contrast was also calculated for the hot micro-spheres positioned between two radioactive IV bags. The percent contrast in hot lesions (Q_H_) is calculated as follows:


(4) $$Q_{H}=\frac{\frac{C_{H}}{C_{B}}-1}{\frac{a_{H}}{a_{B}}-1}\times 100$$

Here, C_H_ and C_B_ represent mean activities in hot and background regions, respectively, while a_B_ and a_H_ represent true activities measured with a dose calibrator (16).

The contrast to noise ratio (CNR) was calculated based on the absolute difference between the mean counts in the hot spheres and the background (for the slice with the maximum hot sphere counts) and was normalized to the standard deviation of the background (SD_B_, Equation 5). This value was used to determine the sphere detectability based on the Rose Criterion (17), which states that objects with CNR < 5 are considered not detectable. Based on this, “pass” or “fail” values for detectability of each sphere in the three lesion-to-background ratio (LBR) acquisitions were reported.


(5) $$CNR\;=\frac{\left|C_{H\;}-\;C_{B}\right|}{SD_{B}}$$

### 2.4 Image quality phantom

**Figure 5:**
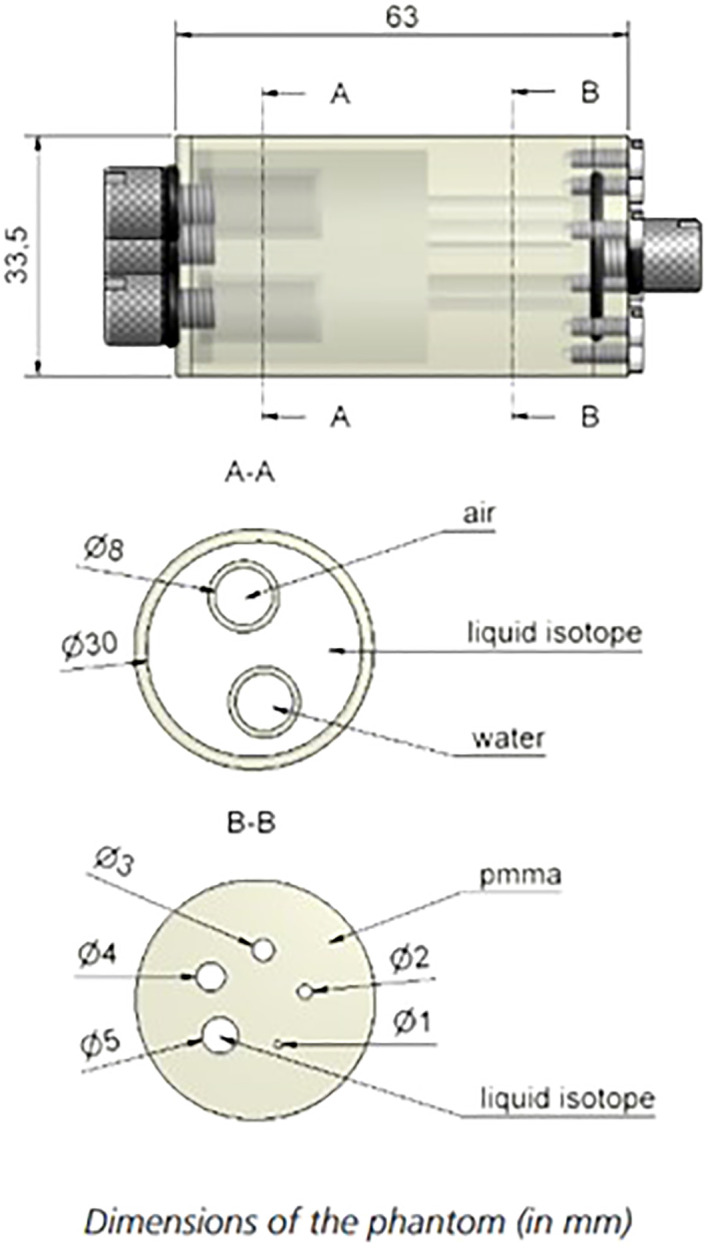
Phantom design of the NEMA NU-4 Image quality phantom. Source: https://www.qrm.de/en/products/micro-pet-iq-phantom/.

NEMA NU 4 image quality phantom (**Figure 5**) contains hot and cold objects of different sizes allowing to complement and verify the measurements of RC as well as to assess image uniformity and the spill-over ratio (SOR) in air and water for the default clinical reconstruction parameters. As shown in **Figure 5**, the phantom volume can be divided into two regions: a solid part with five fillable rods of different diameters to determine the activity recovery coefficients and to assess spatial resolution and partial volume effects of the scanner; and a fillable chamber with two hollow cylinders to be filled with nonradioactive water and air to determine the spill-over ratio in water and air, respectively. A uniform part of this fillable chamber is used for the uniformity and noise measurement, i.e., to determine the mean, maximum and minimum activity concentration and respective %STD similarly to how this was performed for the flood phantom in Section B.

The total activity within the phantom was 1.87 MBq. Acquisitions were performed with the phantom vertically oriented and at a detector separation of 110 mm to accommodate the mounting fixture. The acquisition was calibrated to acquire at least 10 million total events for accurate image reconstruction and processing. The image of the phantom was reconstructed with the default clinical reconstruction parameters, offering additional insights into the performance of attenuation and scatter correction. In line with the NEMA NU-4 protocol (25), the noise within the uniform phantom region serves as an indicator of the imaging system’s signal-to-noise ratio performance. Additionally, the uniformity observed in this region indicates the system’s effectiveness in terms of attenuation and scatter correction. Moreover, measuring activity in the cold regions provides crucial information regarding scatter correction performance.

The uniformity measurement is performed in the central uniform region of the phantom and is based on a cylindrical volume of interest (VOI) with diameter of 22.5 mm and height of 10 mm. Values for the average activity concentration, maximum and minimum voxel values in VOI, and %STD are measured and reported.

The recovery coefficient measurement is performed on the five hot rods using a circular ROI with diameters twice the physical diameter of the rods. The pixel position with the maximum value in each ROI was identified, through which a transverse line profile was drawn. The mean pixel values measured for each profile are divided by the mean activity concentration measured in the uniformity calculation to determine the recovery coefficient for each hot rod in accord with NEMA protocols (25).

The standard deviation of the recovery coefficients per NEMA NU-4 is calculated as follows:


(6) $$\%STD_{RC}\;=\;100\times \sqrt{{\left(\frac{STD_{lineprofile}}{Mean_{lineprofile}}\right)}^{2}\;+\;{\left(\frac{STD_{background}}{Mean_{background}}\right)}^{2}}$$

A cylindrical VOI with diameter of 4 mm and height of 7.5 mm was selected in the central region of the cold (i.e., the air- and water-filled) chambers to assess the accuracy of the applied corrections. Indeed, although both chambers are nonradioactive, scattered annihilation photons and partial volume effect (PVE) due to finite spatial resolution may result in apparent activity in the cold chambers that is characterized by SOR values (26). Explicitly, the SOR was defined as the ratio of the mean in each cold chamber to the mean of the hot uniform area.

The standard deviation of the SOR is calculated as follows:


(7) $$\%STD_{SOR}=100\times \sqrt{{\left(\frac{STD_{cold}}{Mean_{cold}}\right)}^{2}+{\left(\frac{STD_{background}}{Mean_{background}}\right)}^{2}}$$

Both RC and SOR are theoretically limited between 1 and 0.

### 2.5 Clinical imaging demonstration

The clinical performance of the camera is demonstrated through image acquisition in breast cancer patients at varying injected doses of ^18^F-FDG within the framework of a clinical study at the Princess Margaret Cancer Centre of the University Health Network (UHN-PMCC) in Toronto, Canada (27). Participants with a newly diagnosed breast cancer were injected with ^18^F-FDG activities between 37 and 307 MBq (activity was chosen randomly and did not depend on the clinical case). The image acquisition time was fixed for each scan to be 10 minutes. An uptake period of 60 minutes was allocated for each participant prior to image acquisitions. An optional second image set was acquired for patients who opted to return for a subsequent imaging session where the ^18^F activity has decayed to approximately 1/4 of the initial activity (~4 hours post-injection). Image reconstruction was performed using default clinical parameters. For selected images, image segmentation and 3-D lesion volume analysis was performed using an open-source DICOM viewer (3D-Slicer, PET-IndiC).

All clinical images were reviewed in consensus by two fellowship-trained breast radiologists blinded to cancer location. Findings were correlated with histopathology as ground truth. While the pilot clinical study involved 36 patients, and the results can be found in Ref (28), this work specifically presents images of three selected patients. These cases were chosen to emphasize the advantages of organ-targeted PET in addressing clinical challenges that are pertinent to the imaging performance characteristics assessed in this work.

## 3 Results

### 3.1 Spatial resolution & linearity

Reconstructed images of the capillary phantom were used for measurements of spatial resolution. The line cross-sectional profile at 10 different points, evenly distributed along the entire length of the phantom, was approximated by a Gaussian function, and the mean spatial resolution was measured from the average of individual FWHMs. The mean spatial resolution across the in-plane FOV is 2.3 ± 0.1 mm, and the mean Z-axis resolution is 7.9 ± 0.7 mm. The acquisitions were performed at different detector separations between 90 - 135 mm and the results were not dependent on the separation distance.

Spatial linearity measurements were performed on reconstructed images of the linearity phantom shown in **Figure 3**. The mean spatial accuracy in X and Y axes is found to be +/- 0.1 mm. This performance is consistent across and at the edges of the FOV and the results were not dependent on the detector separation distance.

### 3.2 Flood field uniformity

Image uniformity has been assessed in response to uniform exposure across the entire FOV with the flood field phantom. Reconstructed images of flood sources were analyzed for the first and 15^th^ MLEM iteration and uniformity values are summarized in **Table 1**.

**Table 1:** Summary of pixel value uniformity results for the 1^st^ and 15^th^ iterations.

Iterations	Mean	% STD	Min	Max
1	1515	4.1	1228	1717
15	1014	11.7	580	1769

For the 15^th^ iteration used as a default reconstruction parameter, the reconstructed image of the flood field phantom has a uniformity across the FOV of 11.7% standard deviation from the mean value.

### 3.3 Recovery coefficients

**Figure 6:**
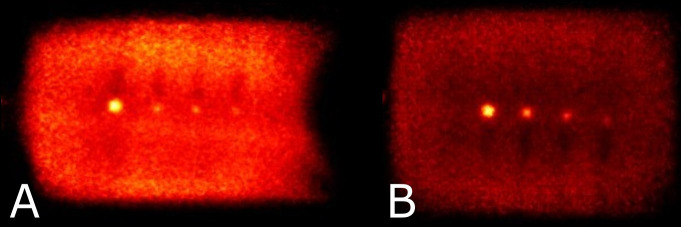
Reconstructed images showing the hot spheres and IV bags at sphere to background activity concentrations of 4:1 **(A)** and 10:1 **(B)**. Note that visual non-uniformity in central regions of the bags is a result of the plastic hot-sphere fixture and a gap in activity at the physical interface between bags.

Reconstructed images of four micro-spheres placed between two 500 mL IV bags (used as uniform background) are shown in **Figure 6** for lesion-to-background activity concentrations of 4:1 and 10:1. Corresponding point-spread functions across the hot spheres are used for calculation of the recovery coefficients from the measured maximum and mean values in each lesion and IV bag background. Recovery coefficients for different sphere sizes across all sphere-to-background ratios are summarized in **Table 2** for comparison with PEM Flex Solo II.

**Table 2:** Summarized recovery coefficients and percent contrast for Radialis PET and two other commercial organ-dedicated PET scanners from phantom experiments.

Sphere Size	8.0 mm Radialis 8.0 mm PEM Flex Solo II 8.4 mm MAMMI PET					4.0 mm Radialis 4.5 mm MAMMI PET		-
Activity Concentration	4:1			10:1		10:1		-
Measured Quantity	RC Relative Mean	RC Relative Max	Absolute RC Max	RC Relative Mean	Percent Contrast (%)	RC Relative Mean	Percent Contrast (%)	Spatial Resolution (mm)
Radialis	2.45	3.27	0.82	4.93	44	2.73	20	2.3
PEM Flex Solo II	1.12	1.40	0.21	-	-	-	-	2.4
MAMMI PET	-	-	-	4.64	42	2.47	17	1.6

Quoted spatial resolution values are provided for comparison (11, 12, 15, 16).

Contrast to noise ratio (CNR) for different sphere sizes across all sphere-to-background ratios are summarized in **Table 3**, along with assessment versus Rose’s Criterion for confidence in assessment of image features (17). These results suggest that sources 6 mm in diameter or larger should receive an accurate contrast assignment for SUV measurement at various lesion-to-background ratios.

**Table 3:** Contrast to Noise ratio for each sphere size and sphere to background activity concentrations with corresponding Rose Criterion assessment.

Sphere Diameters	CNR for 10:1	Rose Criterion	CNR for 4:1	Rose Criterion
8 mm	22.7	PASS	12.8	PASS
6 mm	11.2	PASS	5.7	PASS
5 mm	5.1	PASS	2.8	FAIL
4 mm	2.2	FAIL	0.42	FAIL

### 3.4 Image quality phantom

**Figure 7:**
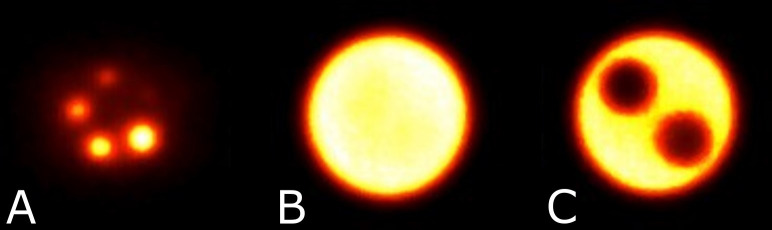
Reconstructed image slices for the NEMA NU-4 image quality phantom displaying the hot rods with diameters 1 - 5 mm for recovery coefficients **(A)**, uniform region **(B)**, and the air and water reservoirs **(C)**.

Transverse images acquired of the NEMA NU-4 Image Quality Phantom are shown in **Figure 7** with visible hot rods (A), uniform region (B), and water and air reservoirs (C).

Uniformity derived as a standard deviation from the mean grey value in the uniform region of NEMA NU-4 image quality phantom is 8.31%. RC and SOR (as well as *%STD_RC_* and *%STD_SOR_* calculated using Equations 6 and 7 respectively) for the organ-targeted Radialis PET camera and PEM Flex Solo II organ-dedicated scanner are presented in **Table 4** and show the expected trend towards full contrast recovery for increasing source sizes. The quoted spatial resolutions, all measured with the same standardized NEMA protocols, are provided to highlight the fact that systems with similar spatial resolution may recover contrast differently in small regions. The results were consistent across the range of detector separations tested (90 - 135 mm).

**Table 4:** Summarized recovery coefficients, spill-over ratio and percent standard deviation for NEMA NU-4 phantom hot rods and cold cylinders for Radialis PET and another commercial organ-dedicated PET scanner (12, 15).

Measured Quantity	RC (%STD)					SOR (%STD)		In-plane Spatial Resolution [mm]
Region	1 mm	2 mm	3 mm	4 mm	5 mm	Air Cylinder	Water Cylinder	-
Radials	0.21 (16)	0.31 (9)	0.53 (10)	0.73 (9)	0.89 (9)	0.30 (19)	0.20 (29)	2.3 ± 0.1
PEM Flex Solo II	0.1 (27)	0.12 (26)	0.22 (14)	0.38 (9)	0.45 (9)	0.64 (11)	0.52 (16)	2.4 ± 0.2

The smallest 1 mm rod in the NEMA NU-4 phantom, although difficult to visualize, has CNR of nearly 2 and shows 21% contrast recovery with a standard deviation of 16%. The largest rod, in comparison, has CNR of greater than 5 and a contrast recovery of 89%. When plotted as a function of sphere size, the recovery coefficient follows a classical “S” shaped sigmoid curve (29).

The larger SOR in air versus water was consistent across sets of measurements. Although it is not discussed in detail here, it was observed that the SOR is highly dependent on the LOR angular filtration. As it will be discussed below, reconstruction software optimization for clinical use requires careful consideration when the aim is to find optimal reconstruction parameters that yield accurate SOR and RC.

### 3.5 Clinical images

**Figure 8:**
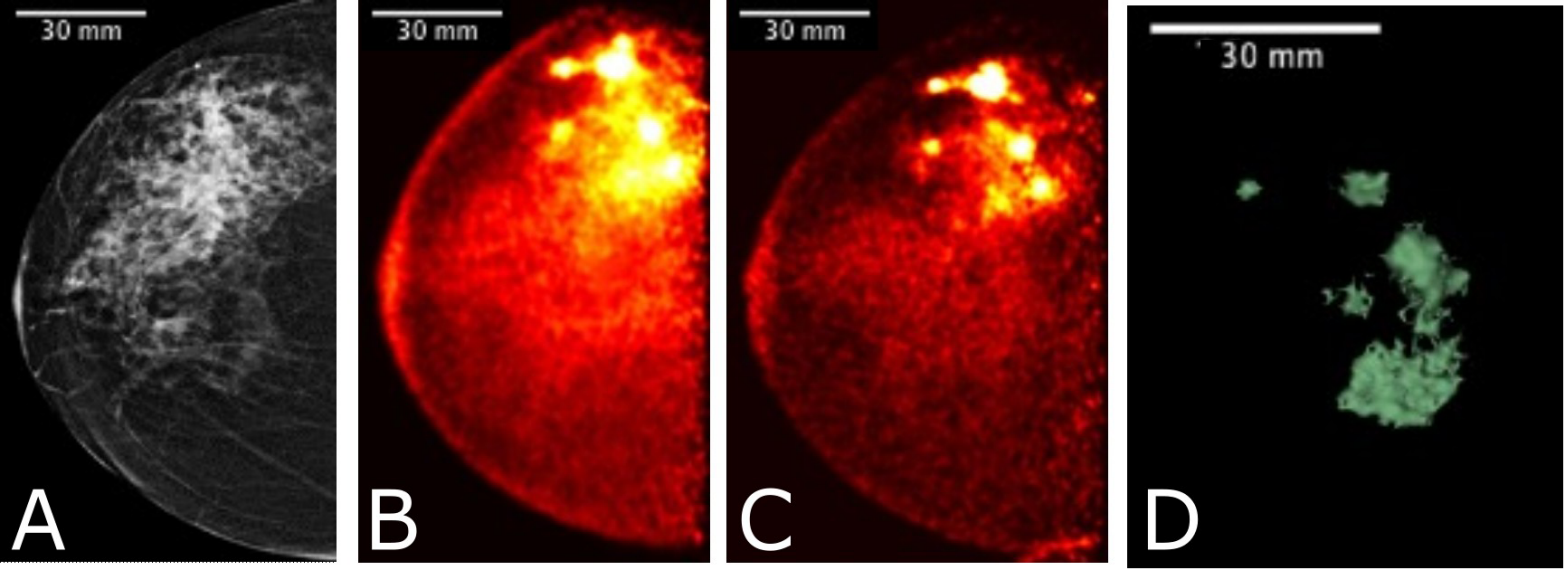
Images acquired for a 61 year-old female with right breast multifocal invasive and *in situ* ductal carcinoma. Images show the same breast in: **(A)** FFDM in the CC plane with extensive distortion; **(B)** 3-D Radialis PET image in the CC plane 1 hour after 178 MBq ^18^F-FDG injection; **(C)** 3-D Radialis PET image in the CC plane where multiple distinct regions of contrast uptake are still evident 4 hours after ^18^F-FDG injection. Mean lesion SUV corrected for lean body mass (SUV_mean, LBM_) is 1.8, with SUV_max, LBM_ equal to 3.4.; **(D)** image of a 3-D volume of different foci generated from Radialis PET in the CC view based upon tissue metabolism across all image slices (12).

**Figure 9:**
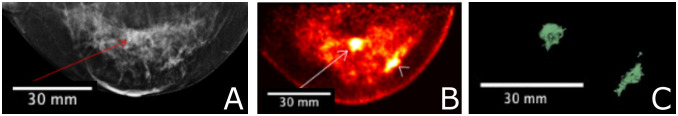
A 56 year-old female with invasive ductal carcinoma and intermediate-grade DCIS underwent FFDM imaging **(A)** with red arrow indicating the site of a primary lesion. Radialis PET image **(B)** acquired 1-hr after injection with 37 MBq ^18^F-FDG and same craniocaudal (CC) view shows two distinct sites of contrast enhancement. The second site (arrowhead) is not detected in mammography. Both sites were confirmed cancerous by histopathology. 3-D volume **(C)** generated from the Radialis PET in the CC view based upon tissue metabolism threshold across all image slices. SUV_mean, LBM_ for the primary lesion is 5.3, with SUV_max, LBM_ equal to 12.2. SUV_mean, LBM_ for the secondary lesion is 5.3, with SUV_max, LBM_ equal to 10.7 (12).

**Figure 10:**
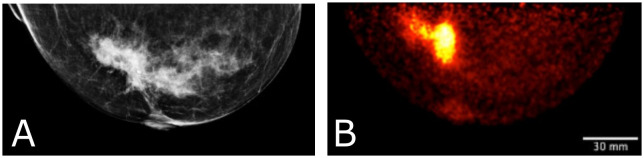
Clinical images of a patient with invasive lobular carcinoma who underwent x-ray mammography **(A)** and Radialis PET imaging 4-hours after radiotracer injection. The 50 year old patient **(B)** received 188 MBq ^18^F-FDG injection and the image shows a craniocaudal (CC) view mammography and PET image. SUV_mean, LBM_ for the lesion in **(B)** is 6.8, with SUV_max, LBM_ equal to 14.9.

Clinical images (**Figures 8**–**10**) are presented here to demonstrate cases where organ-targeted PET imaging is of significant clinical benefit in overcoming challenges in diagnosis, treatment planning, and monitoring response to a therapy.

The presented images in this section are exported from Horos DICOM viewer using the default view conditions for contrast and brightness and PET color look-up table.

**Figure 8** shows a comparison among multimodality images for a multifocal cancer, specifically a FFDM CC view (**Figure 8A**), Radialis organ-targeted PET CC view images (**Figures 8B, C**), and an image of a 3-D reconstruction of multiple foci based on metabolic activity measured with Radialis PET (**Figure 8D**). For the PET scan, 178 MBq of ^18^F-FDG was administrated to the patient and two subsequent imaging sessions (**Figures 8B, C**) were acquired at 1 hour and 4 hours post-injection, with detector separation of 95 mm. The PET images demonstrate ^18^F-FDG uptake in the extensive area that corresponds to the irregular mass detected on digital mammography, and discrimination of multiple foci is still possible even though significant radiotracer decay has occurred. A reconstructed image of 3-D volume of abnormal tissue metabolism is derived from this data set and displayed in **Figure 8D**.

In **Figure 9**, FFDM (**Figure 9A**) is compared to Radialis organ-targeted PET image (**Figure 9B**), and an image of a 3-D volume based on tissue metabolism measured with Radialis PET (**Figure 9C**). A secondary cancerous site is visualized only in the PET image set (arrowhead in **Figure 9B**). The patient was administered 37 MBq of ^18^F-FDG for image acquisition at 1-hour post-injection and images were acquired with detector separation of 120 mm. The images in **Figures 8** and **9** were segmented for analysis and a lean body-mass correction is applied to standardized uptake values quoted for lesions in both patients (30).

**Figure 10** displays a clinical case of an invasive lobular carcinoma (ILC) where distinct sites of enhancement are visible in the organ-targeted PET images and compared against x-ray images. In **Figure 10B**, 188 MBq of ^18^F-FDG was administrated to the patient and images were acquired 4 hours post-injection at a detector separation of 60 mm. The PET image demonstrates localized enhanced ^18^F-FDG uptake at the site of surgical pathology-confirmed ILC. The lean body-mass corrected standardized uptake value is reported for the lesion.

Lesion SUVs for the clinical images presented in **Figures 8** **–** **10** are quoted in **Table 5**, with lean body mass (LBM) correction applied to account for potential overestimation of glucose uptake in obese patients (31).

**Table 5:** Lean body mass corrected standardized uptake values for breast lesions in **Figures 8**-**10**.

Patient		Mean SUV_LBM_ (g/mL)	Maximum SUV_LBM_ (g/mL)	Elapsed Time Post-Injection (hours)
**Figure 8**	Primary lesion	1.4	3.5	1
	Primary lesion	2.2	8.0	4
**Figure 9**	Primary lesion	5.3	12.2	1
	Secondary lesion	5.3	10.7	1
**Figure 10**	Patient with ILC	6.8	14.9	4

## 4 Discussion

Accurate quantitation of SUV holds potential for multifaceted roles in evaluating neoadjuvant treatment effectiveness with 18F-FDG PET. These include: 1) predicting pCR based on pre-treatment (baseline) FDG PET (32, 33); 2) monitoring the decrease in FDG uptake between baseline and interim PET scans (performed during treatment cycles) as predictive of pCR (34–37); and 3) detecting residual primary tumors after NST or identifying exceptional responders in whom breast cancer surgery can be eliminated following NST (38, 39). Here, we focus on selected performance indicators relevant in this context, with special attention to evaluating the recovery coefficient as a major indicator of a PET system’s capabilities for quantitative image assessment.

Ideally, the recovery coefficient approaches unity for active lesions (most malignant tumors in PET) and zero for inactive lesions. However, the measured activity within an active lesion may appear lower than the actual value due to the PVE caused by non-zero spatial resolution. PVE results in reduced contrast assignment and blurred edges around activity boundaries in images. Consequently, small radiation sources tend to spread across the image, leading to a proportional reduction in observed contrast or activity. On the other side, inactive lesions may exhibit apparent spill-in of activity to the cold region. Furthermore, smaller downsized lesions, as a result of successful NST, are more significantly affected by the partial volume effect (40, 41).

While a simplified model suggests that the PVE becomes significant when the size of a tumor is less than two to three times the spatial resolution of the system, our results demonstrate that the issue is more complex. Organ-targeted PET systems with nearly identical spatial resolution may exhibit vastly different recovery coefficients (RCs) for lesions of the same size and activity (see **Table 2**). Therefore, the applicability of PET technology to clinical tasks requiring accurate tumor activity assessment necessitates careful measurement of RCs in different tumor sizes and lesion-to-background ratios, along with other non-standard measurements discussed below.

### 4.1 Spatial resolution & linearity

Spatial resolution performance for the Radialis PET camera is comparable to commercially available organ-dedicated PET scanners (9–11, 16). Measurements presented here confirm our previous point-source results of 2.3 ± 0.1 mm in-plane and Z-axis resolution of 7.9 ± 0.7 mm (12). Since the line source has an inner diameter of 1.2 mm, this is not an intrinsic measurement of resolution, but rather a measurement of finite source size for comparison with whole-body PET.

Reconstructed images of the capillary and linearity phantom demonstrate accurate linear contrast assignment across the entire detector FOV. Intensity peaks from the activity distributions are reconstructed within +/- 0.1 mm of expected locations along both X and Y axes, indicating excellent agreement between expected and measured source locations. Since the linearity phantom extends beyond the FOV, measurements performed to the full extent of the FOV ensures no image distortion at any position within the FOV or at detector edges. Equivalent results are achieved at all four edges of the FOV by reorienting the phantom for measurements, and these findings are consistent with those previously reported for point-source acquisitions (12).

### 4.2 Image uniformity

The uniformity of both tested phantoms (the flood field phantom and NEMA NU-4 Image Quality phantom) deteriorates with increasing MLEM iterations, as expected with iterative maximum likelihood reconstruction algorithms. MLEM is known to amplify noise and potentially induce distortions near edges as iterations increase. However, based on evaluation of phantom and clinical image data, 15 iterations were found to be required for optimal fine detail detection. Therefore, a default setting of 15 iterations is employed for clinical image reconstruction on the organ-targeted PET scanner. Subsequently, image non-uniformity is mitigated by applying optimized MRP filtration within the reconstruction workflow.

### 4.3 Recovery coefficients

**Table 2** summarizes the recovery coefficient and percent contrast (Equation 4) values for the Radialis PET camera and for two other organ-dedicated PET scanners, namely PEM Flex Solo II and MAMMI PET. Quantitative comparison with PEM Flex Solo II was performed at the reported 4:1 activity concentration (11). Despite comparable spatial resolution and detector geometry, all RC values are more than two times better for Radialis PET versus PEM Flex Solo II. In comparison with another organ-dedicated PET scanner, MAMMI PET, which reports nearly 50% higher spatial resolution than Radialis PET (1.6 mm vs. 2.3 mm), the Radialis PET camera has similar yet slightly improved contrast recovery at 10:1 activity concentration, which was the only reported value by MAMMI PET (16). We believe that the improved contrast recovery is a result of greater count efficiency and optimized image reconstruction workflow (12). This claim is subject to further investigation in order to quantify the extent by which count statistics and image reconstruction affect contrast recovery.

The current approach to evaluate PET system performance in terms of confident detectability of small lesions is based on Rose criterion which requires CNR > 5 (17). For 4:1 activity concentration, the Radialis PET camera passes Rose criterion for spheres sized 6 mm and larger. This agrees with theoretical guidelines commonly used in WB PET where the minimum size of spheres that can be measured without underestimation in size and activity is 2.7 times the FWHM spatial resolution of the system (29). However, for 10:1 activity concentration, the Radialis PET camera passes the Rose criterion for spheres smaller than 2.7 times the FWHM (5 mm or 2.17 times the FWHM of 2.3 mm). This indicates that, although theoretical guidelines are largely applicable in WB PET with comparatively low spatial resolution, the ability to reconstruct true activity in high spatial resolution organ-dedicated PET stem from increased count statistics and an ability to apply more rigorous corrections and filtration. Although we do not want to downplay the importance of high spatial resolution in molecular imaging, our results suggest that a system’s contrast recovery capability should be assessed as a significant performance indicator when quantitative assessment of tumor uptake is needed (42).

### 4.4 Image quality phantom

While the suitability of the NEMA NU-4 Image Quality phantom for clinical PET systems is contested in the literature (14), we used it to compare the Radialis PET camera to a commercially available organ-targeted scanner with similar spatial resolution and planar detector architecture, the PEM Flex Solo II. Both scanners visualized hot rods similarly in the NEMA NU-4 phantom, but Radialis PET demonstrated improved RC for 1-5 mm hot rods and lower SOR for air and water-filled cylinders. This suggests that underestimation of reconstructed activity compared to actual activity is not solely due to limited spatial resolution. It also calls into question the universality of a commonly used criterion for the accuracy of reconstructed activity, which links partial volume effect to 2.7-3 times the FWHM of spatial resolution (29, 43, 44) without consideration of other scanner parameters.

Further investigation is needed, but it seems plausible that the higher RC and lower SOR achieved with the Radialis organ-targeted PET system can be attributed to an optimized image reconstruction workflow, a larger field of view, and higher count rate performance. These factors improve the statistical accuracy of measurements, reduce noise, and allow for more rigorous filtration of scattered radiation and random coincidences.

### 4.5 Clinical images

The clinical images presented in this study showcase the potential of organ-targeted PET in breast cancer clinical practice. The results highlight the ability of organ-targeted PET to not only to visualize the spatial distribution of abnormally metabolic tissue but to also quantify its properties in terms of SUV and reconstruct tumor volume based on metabolic activity.

**Figure 8** presents a comparison between FFDM and two Radialis PET images acquired at 1-hour and 4-hours post ^18^F-FDG injection. Despite the changes in image contrast as activity decays post-injection, the radiologist’s visual assessment of multifocal cancers remained unaffected. The multiple regions of enhanced ^18^F-FDG uptake (indicative of multifocal cancers) remained conspicuous even 3 hours after the initial scan and 4 hours from the time of radiotracer administration. Additionally, the 3-D metabolic volume generated from the latter image provides a unique visualization of abnormally metabolic tissue, allowing quantitative tracking of changes in mass volume of abnormally metabolic tissue above a certain threshold.

The results presented in **Figure 8** demonstrate a significant increase in both Mean SUV_LBM_ and Maximum SUV_LBM_ in the course of time after the injection (1.4 vs. 2.2 SUV_LBM, mean_, and 3.5 vs. 8.0 SUV_LBM, max_, **Table 5**) (45). This increase is attributed to differing wash-out mechanisms between cancerous and benign tissues (46). Since SUV_max_ is a significant predictor of tumor detectability, these findings suggest that the scanning protocol may be optimized by increasing the time interval between injection and scanning. The Radialis PET camera is highly sensitive and has improved true coincidence detection (12). This results in a high signal-to-noise ratio, and if the uptake period is longer, the activity decay may not negatively impact image contrast. This enables larger SUV values which may improve the accuracy of tumor assessments.

**Figure 9** shows fundamental advantages of organ-targeted PET in comparison to mammography images for the purpose of both lesion detection and ability for treatment follow-up. The organ-targeted PET image (B) with 37 MBq ^18^F-FDG injection shows two distinct sites of histopathology-confirmed cancerous contrast enhancement, the second of which (arrowhead, **Figure 9B**) is not detected in mammography, even in retrospect. This illustrates the high specificity and sensitivity of Radialis PET imaging in detecting lesions in radiologically dense breast tissue, even at low doses of radiotracer. The measurement of SUV in both the primary and secondary lesions is performed under conditions of ten-times reduced dose, compared to the standard dose of 370 MBq used in PET diagnostic procedures (47).

**Figure 10** illustrates the detection and quantification of invasive lobular carcinoma (ILC) with Radialis organ-targeted PET. ILC is the second most common type of invasive breast cancer, affecting approximately 1 in 10 patients, and its unique biological characteristics make it challenging to detect compared to invasive ductal carcinoma (IDC), the most common type of breast cancer (48, 49). ILC typically exhibits lower FDG uptake compared to IDC (50). This is further compounded by the fact that ILC often presents as diffuse disease with a lack of a clear border, making it more challenging to visualize on PET images. Despite these challenges, Radialis organ-targeted PET images have shown clear enhancement at the sites of surgical pathology-confirmed ILC. The SUV_max_ values correlate well with the lesion size, which is an expected result since FDG uptake may be considered predictive of disease aggressiveness and prognosis for patients with ILC.

We believe that detectability of ILC is due to the overall high sensitivity of Radialis PET and an optimized scanning protocol, which includes a 4-hour time period between injection and scanning. Since various NST’s are applied depending on ILC subtype, with a growing trend toward long-course treatments, organ-targeted PET follow-ups may be of particular utility for accurate staging and treatment adjustments (49).

## 5 Conclusion

The set of measurements performed has revealed a specific peculiarity in high-resolution organ-dedicated PET. We find that the ability to detect and accurately reconstruct true activity in small objects is highly dependent on a broad set of parameters which define PET system performance, and that high spatial resolution alone does not guarantee accurate contrast recovery in small objects. Organ-targeted devices are already understood to exhibit higher spatial resolution than WB PET. Without being tied to other parameters, spatial resolution is not the only metric which defines the clinical utility of a PET system, especially in the context of quantitative measurement of response to therapy.

Our research underscores the significance of the recovery coefficient as a key performance metric for PET systems targeted for small lesion detection, size assessment, and activity uptake quantification. While factors influencing contrast recovery at the lower limits of detection require further evaluation, our findings suggest that focusing solely on improving spatial resolution may not be the most cost-efficient approach. Instead, optimizing the reconstruction algorithm and scanning protocol could be a promising strategy for enhancing PET imaging’s performance, making it a quantitative imaging modality that can accurately measure very low activity values resulting from successful NST.

Although prospective clinical trials are necessary to evaluate the clinical validity of organ-targeted PET for NST, we believe that overall, quantitative organ-targeted PET has the potential to unlock new frontiers in oncology for accurate evaluating early NST response, optimizing its regimen to achieve the maximum pathological effect or identification of non-responders to continuously improve patient outcomes. The emergence of new neoadjuvant therapy options and drugs, coupled with adherence to the principles of precision medical imaging, further highlights the need for quantitative organ-targeted PET.

## Data availability statement

The original contributions presented in the study are included in the article/supplementary material. Further inquiries can be directed to the corresponding author.

## Ethics statement

The studies involving humans were approved by University Health Network Research Ethics Board (18-5029, 8 August 2018) Clinical Trial gov Identifier NCT03520218. The studies were conducted in accordance with the local legislation and institutional requirements. The participants provided their written informed consent to participate in this study.

## Author contributions

BB: Conceptualization, Data curation, Formal analysis, Investigation, Methodology, Software, Validation, Visualization, Writing – original draft, Writing – review & editing. HP: Data curation, Visualization, Conceptualization, Formal analysis, Investigation, Methodology, Project administration, Software, Supervision, Validation, Writing – review & editing. AS: Data curation, Investigation, Software, Validation, Visualization, Writing – review & editing. HM: Investigation, Validation, Data curation, Software, Visualization, Writing – review & editing. MR: Software, Data curation, Investigation, Writing – review & editing. BK: Data curation, Investigation, Software, Validation, Visualization, Writing – review & editing. JS: Data curation, Investigation, Software, Writing – review & editing. VF: Conceptualization, Methodology, Resources, Data curation, Investigation, Validation, Visualization, Writing – review & editing. MW: Conceptualization, Methodology, Resources, Writing – review & editing. OA: Conceptualization, Formal analysis, Methodology, Writing – review & editing. AR: Formal analysis, Conceptualization, Funding acquisition, Investigation, Methodology, Project administration, Resources, Supervision, Validation, Writing – review & editing. OB: Investigation, Software, Writing – review & editing, Conceptualization, Formal analysis, Methodology, Project administration, Resources, Supervision, Validation.

## References

[1] Song D Man X Jin M Li Q Wang H Du Y. A decision-making supporting prediction method for breast cancer neoadjuvant chemotherapy. Front Oncol. (2021) 10:592556. doi: 10.3389/fonc.2020.592556 33469514 PMC7813988

[2] Michalski MH Chen X. Molecular imaging in cancer treatment. EJNMMI. (2011) 38:358–77. doi: 10.1007/s00259-010-1569-z PMC3022114 20661557

[3] Masood S. Neoadjuvant chemotherapy in breast cancers. Women’s Health (London England). (2016) 12:480–91. doi: 10.1177/1745505716677139 PMC5373271 27885165

[4] Ulaner GA. PET/CT for patients with breast cancer: where is the clinical impact? AJR. (2019) 213:254–65. doi: 10.2214/AJR.19.21177 31063423

[5] Vugts G Maaskant-Braat AJG Nieuwenhuijzen GAP Roumen RM Luiten EJ Voogd AC. Patterns of care in the administration of neo-adjuvant chemotherapy for breast cancer. A population-based study. Breast J. (2016) 22(3):316–21. doi: 10.1111/tbj.12568 26945566

[6] Rousset O Rahmim A Alavi A Zaidi H. Partial volume correction strategies in PET. PET Clin. (2007) 2:235–49. doi: 10.1016/j.cpet.2007.10.005 27157875

[7] Hoetjes NJ van Veldenm FH Hoekstra OS Hoekstra CJ Krak NC Lammertsma AA. Partial volume correction strategies for quantitative FDG PET in oncology. Eur J Nucl Med Mol Imaging. (2010) 37(9):1679–87. doi: 10.1007/s00259-010-1472-7 PMC2918791 20422184

[8] Cañadas M Embid M Lage E Desco M Vaquero JJ Pérez JM. NEMA NU 4-2008 performance measurements of two commercial small-animal PET scanners: clearPET and rPET-1. IEEE Trans Nuc Sci. (2011) 58:58–65. doi: 10.1109/TNS.2010.2072935

[9] Gonzalez AJ Sanchez F Benlloch JM. Organ-dedicated molecular imaging systems. IEEE Trans Radiat Plasma Med Sci. (2018) 2:388–403. doi: 10.1109/TRPMS.2018.2846745

[10] Shkumat NA Springer A Walker CM Rohren EM Yang WT Adrada BE. Investigating the limit of detectability of a positron emission mammography device: A phantom study. Med Phys. (2011) 38(9):5176–85. doi: 10.1118/1.3627149 PMC5148033 21978062

[11] MacDonald L Edwards J Lewellen T Haseley D Rogers J Kinahan P. Clinical imaging characteristics of the positron emission mammography camera: PEM flex solo II. J. Nucl Med. (2009) 50(10):1666–75. doi: 10.2967/jnumed.109.064345 PMC2873041 19759118

[12] Stiles J Baldassi B Bubon O Poladyan H Freitas V Scaranelo A. Evaluation of a high-sensitivity organ-targeted PET camera. Sensors. (2022) 22:4678. doi: 10.3390/s22134678 35808181 PMC9269056

[13] Reznik A Bubon O Teymurazyan A. Tileable block detectors for seamless block detector arrays in Positron Emission Mammography. (2017) 4:.

[14] Hallen P Schug D Schulz V. Comments on the NEMA NU 4-2008 standard on performance measurement of small animal positron emission tomographs. EJNMMI Phys. (2020) 7(1):12. doi: 10.1186/s40658-020-0279-2 32095909 PMC7040118

[15] Luo W Anashkin E Matthews CG. Performance evaluation of a PEM scanner using the NEMA NU 4-2008 small animal PET standards. IEEE Trans Nucl Sci. (2010) 57:94–103. doi: 10.1109/TNS.2009.2036847

[16] Moliner L González AJ Soriano A Sánchez F Correcher C Orero A. Design and evaluation of the MAMMI dedicated breast PET. Med Phys. (2012) 39(9):5393–404. doi: 10.1118/1.4742850 22957607

[17] Rose A. Vision: human and electronic. New York: Plenum Press (1973). Opt. Phys. Eng.

[18] Alenius S Ruotsalainen U. Bayesian image reconstruction for emission tomography based on median root prior. Eur J Nucl Med. (1997) 24:258–65. doi: 10.1007/BF01728761 9143462

[19] Øen SK Aasheim LB Eikenes L Karlberg AM. Image quality and detectability in Siemens Biograph PET/MRI and PET/CT systems—a phantom study. EJNMMI Phys. (2019) 6:1–16. doi: 10.1186/s40658-019-0251-1 31385052 PMC6682841

[20] National Electrical Manufacturers Association. NEMA standards publication NU 2-2018: Performance measurements of positron emission tomographs (PET). Rosslyn: National Electrical Manufacturers Association (2018) pp. 41.

[21] Morimoto-Ishikawa D Hanaoka K Watanabe S Yamada T Yamakawa Y Minagawa S. Evaluation of the performance of a high-resolution time-of-flight PET system dedicated to the head and breast according to NEMA NU 2-2012 standard. EJNMMI Phys. (2022) 9(1):88. doi: 10.1186/s40658-022-00518-3 36525103 PMC9758266

[22] Van Sluis J de Jong J Schaar J Noordzij W van Snick P Dierckx R. Performance characteristics of the digital biograph vision PET/CT system. J Nucl Med. (2019) 60(7):1031–6. doi: 10.2967/jnumed.118.215418 30630944

[23] National Electrical Manufacturers Association. NEMA standards publication NU 1-2007: Performance measurements of Gamma Cameras. Rosslyn: National Electrical Manufacturers Association (2007) pp. 1–39.

[24] Nuclear Medicine Committee. Computer-aided scintillation camera acceptance testing. Report Number: AAPM Report No. 9, Authors: A Task Group of the Nuclear Medicine Committee. American Association of Physicists in Medicine Publication, New York (1982). pp. 1–44.

[25] National Electrical Manufacturers Association. NEMA standards publication NU 4-2008: Performance measurements of Small Animal Positron Emission Tomographs. Rosslyn: National Electrical Manufacturers Association (2008) pp 1–23.

[26] Harteveld AA Meeuwis AP Disselhorst JA Slump CH Oyen WJ Boerman OC. Using the NEMA NU 4 PET image quality phantom in multipinhole small-animal SPECT. J Nucl Med. (2011) 52:1646–53. doi: 10.2967/jnumed.110.087114 21849403

[27] Evaluating positron emission mammography imaging of suspicious breast abnormalities. Clinical trial. Identifier: NCT03520218. Available online: https://clinicaltrials.gov/ct2/show/NCT03520218 (accessed on 2 June 2022).

[28] Freitas V Li X Scaranelo A Au F Kulkarni S Chai S. Breast cancer detection using a low-dose positron emission digital mammography system. Radiol: Imaging Cancer. (2024) 6:230020. doi: 10.1148/rycan.230020 PMC10988332 38334470

[29] Prieto E Martí-Climent JM Arbizu J Garrastachu P Domínguez I Quincoces G. Evaluation of spatial resolution of a PET scanner through the simulation and experimental measurement of the recovery coefficient. Comput Biol Med. (2010) 40(1):75–80. doi: 10.1016/j.compbiomed.2009.11.002 19959163

[30] Keramida G Hunter J Dizdarevic S Peters AM. The appropriate whole-body index on which to base standardized uptake value in 2-deoxy-2-[18F] fludeoxyglucose PET Brit. J Radiol. (2015) 88(1052):20140520. doi: 10.1259/bjr.20140520 26081445 PMC4651382

[31] Sugawara Y Zasadny KR Neuhoff AW Wahl RL. Reevaluation of the standardized uptake value for FDG: variations with body weight and methods for correction. Radiology. (1999) 213:521–5. doi: 10.1148/radiology.213.2.r99nv37521 10551235

[32] Oliveira C Oliveira F. Vaz SC Marques HP Cardoso Fátima. Prediction of pathological response after neoadjuvant chemotherapy using baseline FDG PET heterogeneity features in breast cancer. Br J Radiol. 96(1146):20220655. doi: 10.1259/bjr.20220655 PMC10230392 36867773

[33] Bouron C Mathie C Morel O Seegers V Guillerminet C Lacoeuille F. Correlation between baseline 18F-FDG PET/CT features and pathological complete response after neoadjuvant chemotherapy in early triple negative breast cancer. Médecine Nucléaire. (2021) 45:135–41. doi: 10.1016/j.mednuc.2021.01.007

[34] Pahk K Kim S Choe JG. Early prediction of pathological complete response in luminal B type neoadjuvant chemotherapy-treated breast cancer patients: Comparison between interim 18 F-FDG PET/CT and MRI. Nucl Med Commun. (2015) 36:887–91. doi: 10.1097/MNM.0000000000000329 25932536

[35] Chen S Ibrahim NK Yan Y Wong ST Wang H Wong FC. Complete metabolic response on interim 18F-fluorodeoxyglucose positron emission tomography/computed tomography to predict long-term survival in patients with breast cancer undergoing neoadjuvant chemotherapy. Oncologist. (2017) 22:526–34. doi: 10.1634/theoncologist.2016-0334 PMC5423520 28377466

[36] Groheux D Sanna A Majdoub M de Cremoux P Giacchetti S Teixeira L. Baseline tumor 18F-FDG uptake and modifications after 2 cycles of neoadjuvant chemotherapy are prognostic of outcome in ER+/HER2- breast cancer. J Nucl Med. (2015) 56:824 – 31. doi: 10.2967/jnumed.115.154138 25883123

[37] Kazerouni AS Peterson LM Jenkins I Novakova-Jiresova A Linden HM Gralow JR. Multimodal prediction of neoadjuvant treatment outcome by serial FDG PET and MRI in women with locally advanced breast cancer. Breast Cancer Res. (2023) 25(1):138. doi: 10.1186/s13058-023-01722-4 PMC10636950 37946201

[38] Romeo V Accardo G Perillo T Basso L Garbino N Nicolai E. Assessment and prediction of response to neoadjuvant chemotherapy in breast cancer: A comparison of imaging modalities and future perspectives. Cancers. (2021) 13:3521. doi: 10.3390/cancers13143521 34298733 PMC8303777

[39] Kuerer HM Rauch GM Krishnamurthy S Adrada BE Caudle AS DeSnyder SM. A clinical feasibility trial for identification of exceptional responders in whom breast cancer surgery can be eliminated following neoadjuvant systemic therapy. Ann Surg. (2018) 267(5):946–51. doi: 10.1097/SLA.0000000000002313 PMC6051523 28549010

[40] Soret M Bacharach SL Buvat I. Partial-volume effect in PET tumor imaging. J Nucl Med. (2007) 48:932–45. doi: 10.2967/jnumed.106.035774 17504879

[41] Cysouw MCF Kramer GM Schoonmade LJ Boellaard R de Vet HCW Hoekstra OS. Impact of partial-volume correction in oncological PET studies: a systematic review and meta-analysis. Eur J Nucl Med Mol Imaging. (2017) 44(12):2105–16. doi: 10.1007/s00259-017-3775-4 PMC5656693 28776088

[42] Srinivas SM Dhurairaj T Basu S Bural G Surti S Alavi A. A recovery coefficient method for partial volume correction of PET images. Ann Nucl Med. (2009) 23(4):341–8. doi: 10.1007/s12149-009-0241-9 19367446

[43] Meechai T Tepmongkol S Pluempitiwiriyawej C. Partial-volume effect correction in positron emission tomography brain scan image using super-resolution image reconstruction. Br J Radiol. (2015) 88:20140119. doi: 10.1259/bjr.20140119 25492553 PMC4614236

[44] Vaidya JS Massarut S Vaidya HJ Alexander EC Richards T Caris JA. Rethinking neoadjuvant chemotherapy for breast cancer. BMJ. (2018) 360:j5913. doi: 10.1136/bmj.j5913 29326104

[45] Beaulieu S Kinahan P Tseng J Dunnwald LK Schubert EK Pham P. SUV varies with time after injection in 18F-FDG PET of breast cancer: characterization and method to adjust for time differences. J Nucl Med. (2003) 44(7):1044–50. 12843218

[46] Macdonald LR Hippe DS Bender LC Cotter EW Voria PR Hallam PS. Positron emission mammography image interpretation for reduced image count levels. J Nucl Med. (2016) 57:348–54. doi: 10.2967/jnumed.115.165787 26635337

[47] Boellaard R Delgado-Bolton R Oyen WJG Giammarile F Tatsch K Eschner W. FDG PET/CT: EANM procedure guidelines for tumour imaging: Version 2. 0 Eur J Nucl Med Mol Imaging. (2015) 42:328–54. doi: 10.1007/s00259-014-2961-x PMC4315529 25452219

[48] Pestalozzi BC Zahrieh D Mallon E Gusterson BA Price KN Gelber RD. Distinct clinical and prognostic features of infiltrating lobular carcinoma of the breast: combined results of 15 International Breast Cancer Study Group clinical trials. J Clin Oncol. (2008) 26(18):3006–14. doi: 10.1200/JCO.2007.14.9336 18458044

[49] Mukhtar RA Hoskin TL Habermann EB Day CN Boughey JC. Changes in management strategy and impact of neoadjuvant therapy on extent of surgery in invasive lobular carcinoma of the breast: analysis of the national cancer database (NCDB). Ann Surg Oncol. (2021) 28(11):5867–77. doi: 10.1245/s10434-021-09715-3 PMC8460506 33687613

[50] Jung NY Kim SH Choi BB Kim SH Sung MS. Associations between the standardized uptake value of (18)F-FDG PET/CT and the prognostic factors of invasive lobular carcinoma: in comparison with invasive ductal carcinoma. World J Surg Oncol. (2015) 13:113. doi: 10.1186/s12957-015-0522-9 25889560 PMC4371618

